# Photo-induced Doping in GaN Epilayers with Graphene Quantum Dots

**DOI:** 10.1038/srep23260

**Published:** 2016-03-18

**Authors:** T. N. Lin, M. R. Inciong, S. R. M. S. Santiago, T. W. Yeh, W. Y. Yang, C. T. Yuan, J. L. Shen, H. C. Kuo, C. H. Chiu

**Affiliations:** 1 Department of Physics and Center for Nanotechnology, Chung Yuan Christian University, Chung-Li, Taiwan; 2 Department of Photonic and Institute of Electro-Optical Engineering, National Chiao Tung University, Hsin-Chu, Taiwan; 3 Department of Electronic Engineering, Chung Yuan Christian University, Chung-Li, Taiwan; 4 Adavanced Optoelectronic Technology Inc., Hsin-Chu, Taiwan

## Abstract

We demonstrate a new doping scheme where photo-induced carriers from graphene quantum dots (GQDs) can be injected into GaN and greatly enhance photoluminescence (PL) in GaN epilayers. An 8.3-fold enhancement of PL in GaN is observed after the doping. On the basis of time-resolved PL studies, the PL enhancement is attributed to the carrier transfer from GQDs to GaN. Such a carrier transfer process is caused by the work function difference between GQDs and GaN, which is verified by Kelvin probe measurements. We have also observed that photocurrent in GaN can be enhanced by 23-fold due to photo-induced doping with GQDs. The improved optical and transport properties from photo-induced doping are promising for applications in GaN-based optoelectronic devices.

GaN-based semiconductors have attracted considerable attention due to their great advantages of low energy consumption, high brightness, and long lifespan. In recent years, advanced growth techniques of GaN-based semiconductors have led to the achievements of light emitting diodes (LEDs) and laser diodes with high brightness and high reliability^1^,^2^,^3^,^4^,^5^. For further developments of GaN-based devices, enhanced luminescence and photoconductivity properties in GaN are necessary. Graphene, consisting of a single sheet sp^2^-hybridized carbon with a hexagonal lattice, is a two-dimensional planar material. Due to its fascinating physical properties and potential applications, graphene has been extensively investigated^6^,^7^,^8^. Over the last several years, the preparation and applications of graphene/semiconductor composite have been conducted^9^,^10^,^11^. At the graphene/semiconductor interface, charge carriers are able to transfer from many semiconductors to graphene because the work function of graphene (approximately 4.7 eV) is larger than that of most semiconductors^9^,^10^,^11^,^12^. However, such a carrier transfer from graphene to semiconductors causes a reduction in the photoluminescence (PL) or photoconductivity in semiconductors, which is undesirable in optoelectronic applications^11^. Graphene quantum dots (GQDs), with lateral dimensions less than 100 nm, are a new class of graphene nanostructures. GQDs exhibit fascinating properties, such as low toxicity, biocompatibility, and strong PL. The work function of GQDs is different from that of graphene because there are plenty of oxygen-related functional groups on the surface of GQDs. It has been reported that the work function of graphene moieties can be greatly modulated by oxygen and/or oxygen functionalities^13^,^14^. Recently, the work function of GQDs has been investigated theoretically and experimentally^13^,^15^,^16^,^17^. The calculated work function of GQDs has been found to be approximately 3.6–3.8 eV using a tight binding model^13^,^15^. Using Mott-Schottky plots, cyclic voltammetry, and ultraviolet photoelectron spectroscopy, the work functions of GQDs have been measured to be 3.47, 3.55, and 3.5–3.7 eV, respectively^13^,^16^,^17^. Therefore, the work function of GQDs lies in the range of 3.5–3.8 eV, which is smaller than that of graphene and many semiconductors^12^. Due to the work function difference, photo-induced carrier injection can occur from GQDs to semiconductors, leading to an increase in the carrier concentration in semiconductors. GQDs can thus be a dopant to donate carriers, which may have promising applications in optoelectronic devices.

Many new types of doping have recently been proposed, and charge transfer between dopant molecules and host materials has modulated the optical and electrical properties of host materials^18^,^19^,^20^. For example, photo-induced doping in graphene/BN heterostructures, analogous to the modulation doping in semiconductor heterojuctions, has been demonstrated and can be used to create inhomogeneous doping in graphene devices^18^. Molecular chemical doping with the injection and extraction of electrons has been implemented in monolayer MoS_2_ for modifying its optical and electrical properties^19^. Additionally, the incorporation of hole dopants into single-walled carbon nanotubes (SWCNTs) has created a relaxation channel from dark excitons to trions, indicating that a small amount of doping can modulate the optical properties of SWCNTs^21^. In this work, a new doping scheme is proposed, where the photo-induced charge carriers from GQDs (dopants) can be transferred into GaN (host crystals) as shown in Fig. 1. An enhancement of PL intensity and photocurrent in GaN is observed in GQD-doped GaN. On the basis of PL decay dynamics and Kelvin probe measurements, we discuss the mechanism that causes the carrier transfer from GQDs to GaN. This study indicates that the incorporation of GQDs on GaN can greatly improve the PL and photoconduction properties of GaN, which is important for applications of GaN-based devices.

## Results

### Characterizations of graphene quantum dots

**Figure 2:**
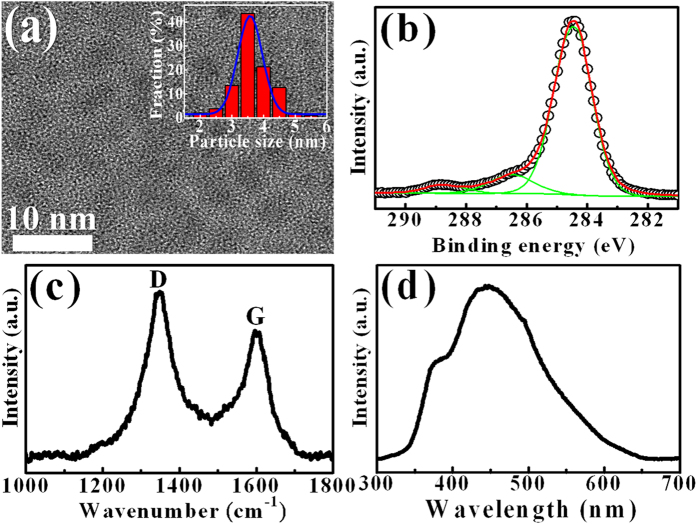
(**a**) TEM image of the GQDs. The inset shows the size distribution of the GQDs. The solid line indicates the Gaussian fit. (**b**) C1s XPS spectrum of GQDs. (**c**) Raman spectrum of GQDs. (**d**) PL spectrum of GQDs at 260 nm excitation wavelength.

The typical TEM image of the synthesized GQDs is shown in Fig. 2a, revealing a monodispersed distribution. The size distribution is displayed in the inset, obeying a Gaussian curve. An average size and a full width at half maximum (FWHM) was determined to be ~3.5 nm and ~3 nm for GQDs, respectively. Figure 2b shows the C1s XPS spectrum of the synthesized GQDs, which is deconvoluted into three peaks. These three peaks at 284.4, 286.1, and 288.7 eV binding energies correspond to the sp^2^ aromatic carbon (C = C), epoxy groups (C-O), and carboxyl groups (C(O)-OH), respectively^22^,^23^. Similar binding energies of GQDs determined from XPS spectra have been reported^22^,^23^. For examining the graphenic structure of the materials, Raman spectroscopy was carried out. Figure 2c displays the Raman spectrum of the as-prepared GQDs. Two bands were clearly observed: the D band at ~1348 cm^−1^ and G band at ~1597 cm^−1^, representing the defect-induced breathing mode of aromatic rings and the optical E_2g_ phonons at the Brillouin zone center, respectively^12^,^24^. In general, the intensity ratio of the D and G bands illustrates a measure of disorder degree, which is inversely proportional to the average size of the sp^2^ crystallite region. The average crystallite size of the sp^2^ domain *L*_*a*_ in the graphene structure can be estimated by^12^,^24^:


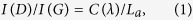


where *C*(λ) is an empirical constant related to the wavelength of the excitation laser, *I*(*D*) is the intensity of the D band, and *I*(*G*) is the intensity of the G band. The calculated *L*_*a*_ was estimated to be ~3.6 nm for GQDs, which is comparable to the GQDs size determined from TEM.

The PL spectrum of the synthesized GQDs is displayed in Fig. 2d when excited at a wavelength of 260 nm. A blue PL band that peaks at ~450 nm was observed. The PL of GQDs has been investigated recently and it generally exhibits a blue or green peak^25^,^26^,^27^,^28^,^29^,^30^,^31^,^32^. The blue peak of GQDs has been assigned to intrinsic free zigzag sites with carbon crystalline structures, carbene-like triplet states, or band-to-band π*-π transitions^25^,^28^,^29^. The green peak has been associated with the extrinsic defect (surface) states or the electronic transition from lowest unoccupied molecular orbits (LUMOs) to highest occupied molecular orbits (HOMOs)^26^,^27^. However, the exact luminescence mechanism of GQDs is an open question and still under investigation. The PL peak in GQDs can be affected by the preparation procedure of the GQDs^30^. This could be attributed to the complex PL origins of GQDs, which depends on their size, shape, edge type (zigzag and armchair edges), surface configuration, and solvent^30^,^32^. The PL spectra of GQDs with different GQD concentrations are displayed in Figure S1. The wavelength of the PL peak in GQDs remains unchanged as the concentration increases from 0.3 to 0.9 mg/ml. It has been reported that a red shift of PL peak may occur in the PL of GQDs as the GQD concentration increases, attributing to the self-assembled J-type aggregation under restrained π-π interactions^33^. The invariance of the peak wavelength of PL in our case indicates that no aggregation occurs in our GQDs. The PL peak of GQDs is red-shifted as the excitation wavelength increases (Figure S1). Similar excitation wavelength dependence of the PL properties has been reported for the GQDs synthesized from top-down methods^30^. This excitation dependence behavior may originate from the heterogeneity of GQDs in size, emission sites, functional groups, and defects^30^,^31^,^32^.

### PL enhancement

**Figure 3:**
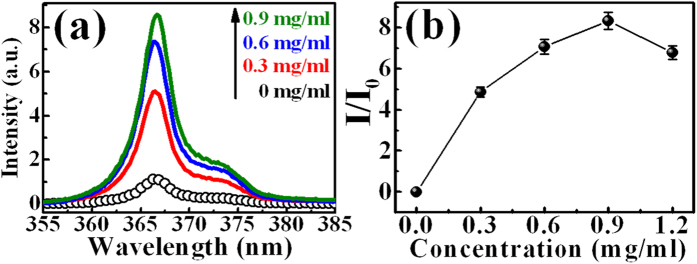
(**a**) PL spectra of the GQD-doped GaN with different GQD concentrations. (**b**) The PL intensity ratio of the GaN with GQDs to that of the bare GaN as a function of the GQD concentration.

The PL spectrum of the GaN epilayer is displayed as the open circles in Fig. 3a. The spectrum shows a main peak at a wavelength of 367 nm, which was assigned to the band-edge emission in GaN^34^. Changes of the PL intensity after doping of GQDs with different concentrations are shown as solid lines in Fig. 3a. An enhancement of PL intensity in GaN occurs after doping with GQDs. The PL peak intensity of the pure GQDs with a concentraion of 2.4 mg/ml is about one order smaller than that of the GaN, revealing that the enhanced PL in GaN is not originated from the direct emission of GQDs. (Figure S2) Fig. 3b shows the PL intensities in GaN versus the GQD concentration, indicating the increase of PL intensity with increasing GQD concentration. The maximum PL intensity occurs at the GQD concentration of 0.9 mg/ml, revealing an enhancement factor of 8.3. The enhancement of PL intensity under photoinduced doping may make the GaN particularly useful for the further applications in optical devices such as light emitting diodes (LEDs), ultraviolet lasers, electroabsorption modulators, optical switches, and solar cells.

### Doping mechanism

**Figure 4:**
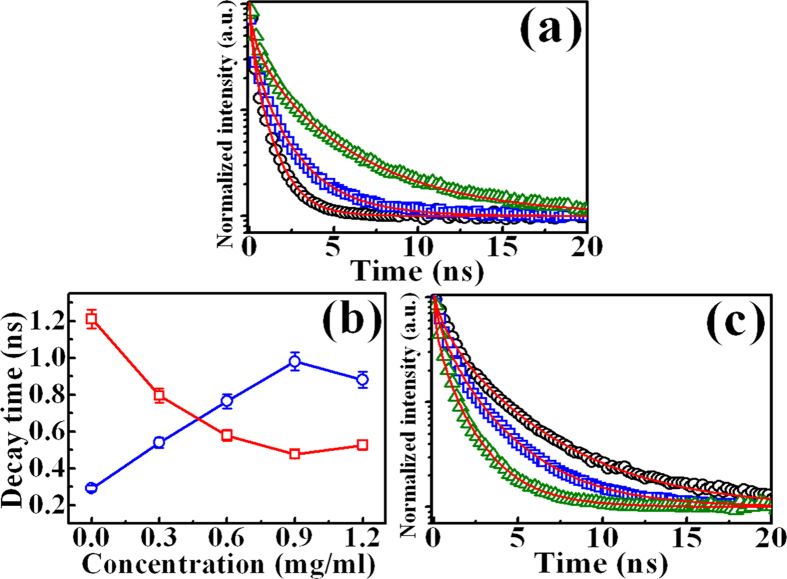
(**a**) The PL decay profiles of GQD-doped GaN with different GQD concentrations: 0 (circles), 0.3 (squares), and 0.9 mg/ml (triangles). (**b**) The PL decay time of GaN (open circles) and GQDs (open squares) as a function of the GQD concentration. (**c**) The PL decay profiles of the GQDs after doping with different GQD concentrations: 0 (circles), 0.3 (squares), and 0.9 mg/ml (triangles). The solid lines display the fitted curves using Eq. (2).

To further find out the doping mechanism, time-resolved PL measurements of bare GaN and the GQD-doped GaN were investigated. The PL decay profile of bare GaN epilayers monitored at the peak wavelength of 367 nm is shown as the open circles in Fig. 4a. The open squares and triangles in Fig. 4a shows the PL decay profiles of GaN after the doping of GQDs with the concentration of 0.3 and 0.9 mg/ml, respectively. The PL decay transients in the GQD-dope GaN decay less as compared to the bare GaN. The PL decay curves can be fitted by the stretched exponential function:


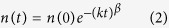


where *n*(*t*) is the carrier density and *k* is the decay rate of carriers and *β* is a dispersive exponent. The fitted results are shown as a solid line in Fig. 4a which are in good agreement with experiments. All the fitting parameters used are listed in Table 1. The average decay time in the stretched exponential function is described by^35^:


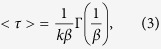


where Γ is the Gamma function. The open circles in Fig. 4b shows the obtained PL decay times of GaN after doping as a function of the GQD concentration. It was found that the PL decay time in GaN increases with increasing amount of GQDs concentration.

The PL decay profiles of the dopants (GQDs) before and after doping in GaN monitored at the peak wavelength of 450 nm are shown in Fig. 4c. The PL decay transients of the GQDs after doping decay more as compared with that in bare GQDs. The PL decay curves in Fig. 4c were also fitted by the stretched exponential function and the PL decay times were determined using Eq. (2) and (3). All the fitting parameters and the PL decay times are listed in Table 2. The open squares in Fig. 4b displays the PL decay time of GQDs after deposition on GaN as a function of the GQD concentration. The PL decay times of the GQDs decreases with increasing the amount of the GQD concentration.

From the PL dynamics of GaN and GQDs (dopants), we found that the increase of the PL decay times of GaN is in parallel to the decrease of the PL decay times of GQDs as shown in Fig. 4b. This observation implies that carrier transfer may take place between GaN and GQDs, leading to the above changes in the PL decay time. To find out the origin of carrier transfer, the work function measurements by the Kelvin probe were carried out. The contact potential difference (*V*_*CPD*_) between the sample and tip can be associated with the difference in work function between them which is described by:


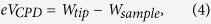


where *W*_*tip*_ and *W*_*sample*_ are work functions of the tip and sample, respectively. With calibration of *W*_*tip*_ and measurement of *V*_*CPD*_, the sample work function can be determined. Using Eq. (4), the work functions of GQDs and GaN were determined to be 3.64 ± 0.03 eV and 4.38 ± 0.03 eV, respectively. The obtained work functions are in comparable with the reported values^36^,^37^.

**Figure 5:**
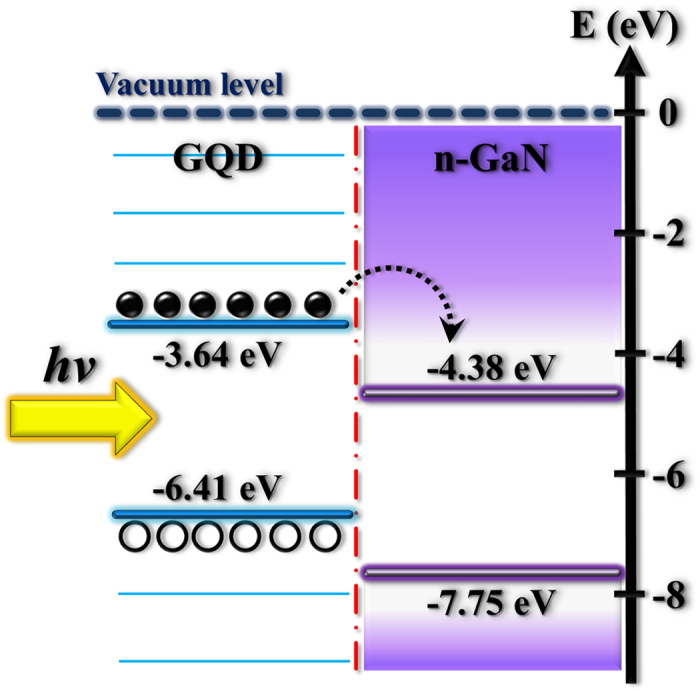
Energy band diagram of the GQD-doped GaN.

From the experimental results in PL, time-resolved PL, and work-function measurements, the carrier transfer owing to photoinduced-doping can be explained as follows. For the photoexcitation with photon energy greater than the band-gap energy of GQDs, the carriers are generated in the GQDs. The photoinduced carriers can efficiently transfer from GQDs to GaN through the GQD/GaN interface since the work function of GQDs (3.64 eV) is smaller than that of GaN (4.38 eV), as shown in Fig. 5. The increased carrier density in GaN, originated from the carrier transfer from GQDs to GaN, leads to an enhancement of the PL intensity and PL decay time in GaN. With the higher GQD doping more carrier densities can be transferred from GQDs to GaN, reflecting more enhanced PL intensity in GaN, as shown in Fig. 3b. The PL decay time in GaN is also increased more under the GQD doping with higher concentrations (Fig. 4b). On the other hand, the carrier transfer from GQDs to GaN leads to a loss of the photoinduced carrier density in GQDs. With the higher GQD doping more carrier loss in GQDs produces a pronounced decrease of the PL decay time in GQDs, as shown in Fig. 4c. Thus, the concentration of GQDs play an important role in the GQD doping since it can control the PL intensity and decay time in GaN through the carrier transfer. Similar studies of carrier transfer between GQDs and semiconductors have been recently reported^12^,^38^.

It is well known that surface plasmon coupling can be a mechanism to enhance the spontaneous emission of a semiconductor. The plasmon-induced PL enhancement in semiconductors has been reported by introduction of graphene or reduced graphene oxide^39^,^40^,^41^,^42^. In the surface plasmon coupling, the spontaneous emission and recombination rate is enhanced when the emission energy of a semiconductor resonantly corresponds to the energy of surface plasmon. For the surface plasmon coupling, the induced PL enhancement is associated with a semiconductor in either absorption or emission processes^41^,^43^. Figure S3 displays the absorbance of GaN without and with the introduction of GQDs. The absorbance of GaN decreases after the introduction of GQDs, indicating no plasmon-induced absorption enhancement. Also, the plasmon enhancement in the emission process will lead to an increase in the spontaneous emission rate, i.e., a decrease of a PL decay time^44^,^45^. Figure 4a shows the PL decay time in GaN increases after the introduction of GQDs, opposite to expectation of the plasmon enhancement in the emission process. (Furthermore, the PL enhancement from plasmon effect depends on the excitation wavelength, which will cause a peak shift in the PL spectrum^44^. However, no peak shift occurs in our PL enhancement in GaN.) Therefore, according to the above arguments, the mechanism based on the surface plasmon coupling should be ruled out from the interpretation of PL enhancement in GaN.

Another possible PL enhancement in semiconductors is fluorescence resonance energy transfer (FRET), in which the electronic excitation energy of donors is transferred to acceptors via nonradiative dipole-dipole interaction^46^. The occurrence of FRET requires an overlap between the emission spectrum of donors and the absorption spectrum of acceptors, and the FRET efficiency has a strong dependence on the donor-acceptor separation. In our case, GaN and GQDs are the donor and the acceptor, respectively, because the band-gap energy of GaN (~3.38 eV) is larger than that of GQDs (~2.75 eV). According to the FRET mechanism, only the PL of GQDs can be enhanced since the electronic energy is transferred from donors (GaN) to acceptors (GQDs). This is opposite to the observation of PL enhancement of GaN in the GQD/GaN composite (Fig. 3a). Thus, FRET from GaN to GQDs cannot be used to explain the PL enhancement in GaN.

### Photocurrent enhancement

**Figure 6:**
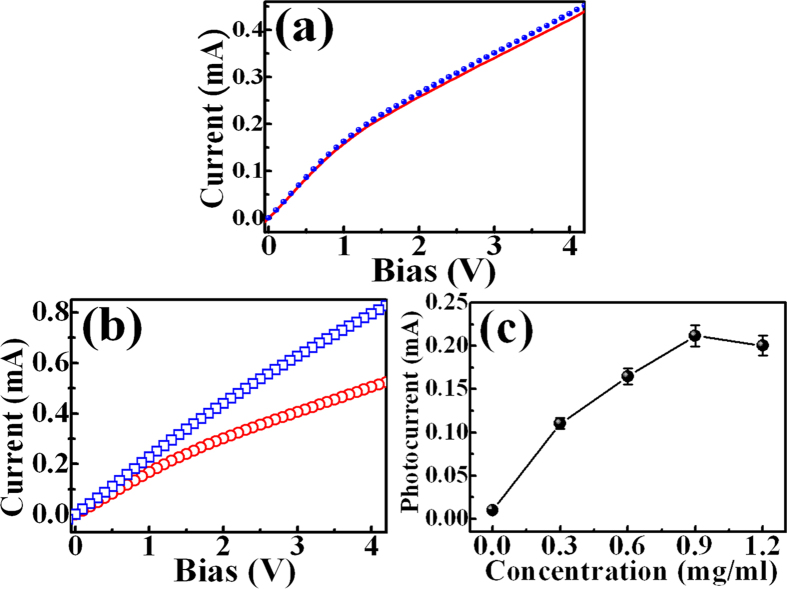
The dark (the dashed line) and light (the solid line) current-voltage curves of the (**a**) bare GaN and (**b**) GQD-doped GaN. (**c**) The photocurrent of the GQD-doped GaN as a function of the GQD concentration.

The photoinduced doping with GQDs is potentially applicable in the GaN-based optoelectronic devices such as LEDs and photodetectors. Thus, it is desirable to investigate the transport property of the GQD-doped GaN. The line and solid circles in Fig. 6a shows the I–V characteristics of the bare GaN in the dark and under illumination of a 260-nm laser, respectively. Little increase of conductivity due to photogenerated carriers is observed. The obtained photocurrent (Δ*I* = *I*_light_ − *I*_dark_) was found to be 0.012 mA under a bias voltage of 4 V. On the other hand, the open circles and squares in Fig. 6b show the *I–V* characteristics of the GQD-doped GaN in the dark and under illumination, respectively. Obviously, the GaN after doping reveals a larger photocurrent (0.28 mA) than the bare GaN under the same condition. Figure 6c shows the photocurrent of the GQD-doped GaN as a function of the GQD concentration. The magnitude of photocurrent in GaN is proportional to the GQD concentration. At the concentration of 0.9 mg/ml, more than 23-fold of magnitude in photocurrent is found at a bias at 4V. The increased photocurrent in GaN due to GQDs is also attributed to an increase of the photoinduced carriers transferring from dopants (GQDs) to GaN. More GQD concentration would contribute to more light absorption and carrier density, leading to a larger photocurrent in the GaN layer as shown in Fig. 6c.

**Figure 7:**
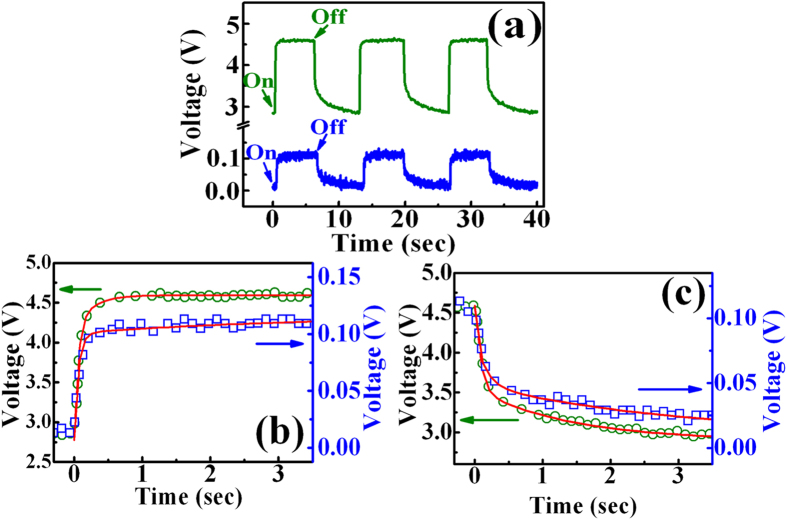
(**a**) Photoresponse of the bare GaN and the GQD-doped GaN at a bias current of 0.8 mA under 260 nm illumination. Time response of photovoltage rise and decay for the (**b**) bare GaN and (**c**) GQD-doped GaN. The solid lines show fits to the exponential equations.

The blue and green line in Fig. 7a shows the transient photocurrent responses of the bare GaN and the GQD-doped GaN during the switching of 260-nm laser at a current of 0.8 mA, respectively. The photovoltage increases when the light is turned on and decreases to their initial values after turning off the light. Figure 7b,c displays the time response of photovoltage rise (decay) for the bare GaN and the GQD-doped GaN under the switching of 260-nm laser, respectively. The rise and decay time constants can be described by I(t) = I_dark_ + A exp(t/τ_r1_) + B exp(t/τ_r2_) and I(t) = I_dark_ + A exp(−t/τ_d1_) + B exp(−t/τ_d2_) respectively, where I_dark_ is the dark voltage, A and B are scaling constants, and τ_r_ (τ_d_) is the rise (decay) time constant^47^. The solid lines are a fit to the experimental results and the fitting parameters are listed in Table 3. The rise (decay) time of photovoltage in the GQD-doped GaN is shorter than that of the bare GaN, revealing an improvement of the photoresponse time. These results indicate that GQDs can be efficient dopants for donating carriers for improving optoelectronic properties in GaN, which may be crucial to their applications in GaN-based devices.

## Discussion (Conclusion)

In summary, the effect of the photoinduced doping with GQDs has been demonstrated on GaN epilayers. We observe an eight-fold enhancement of the PL intensity in GaN epilayers at the GQDs concentration of 0.9 mg/ml. On the analysis of PL dynamics and the Kelvin probe measurements, we suggest that the efficient carrier transfer from GQDs to GaN is responsible for the PL enhancement. We also found that the photoinduced doping with GQDs leads to a 23-fold enhancement of photocurrent in GaN under UV illumination, which is promising for potential applications in the GaN-based devices.

## Methods

### Preparation of GQDs and n-GaN epilayers

The GQD dopants were synthesized by the pulse laser ablation method^12^. The graphene flakes for synthesis of GQDs were purchased from *Graphene Supermarket* (USA). We used an OPO laser delivering 10 ns pulses at 415 nm with 10 Hz repetition rates as the laser excitation source. The graphene flakes suspended in methanol were placed in a quartz cell and irradiated to the laser pulses on a rotational stage (an angular velocity of 80 rpm). The laser radiation was controlled under the fluence of 2.58 J/cm^2^ for 5 min. The suspension product in methanol was filtered using the syringe filter (Millipore, 0.22 μm pore size) after pulse laser ablation. The n-GaN epilayers were grown by metal-organic chemical vapor deposition on a sapphire substrate. The epitaxial structure is composed of a 30 nm GaN nucleation layer, a 2 μm un-doped GaN buffer layer, and a 1 μm Si-doped *n*-GaN layer (doping concentration = 5 × 10^18^ cm^−3^). In the growth, trimethylgallium (TMGa), ammonia (NH_3_), and silane (SiH_4_) were used as gallium, nitrogen, and silicon sources, respectively.

### Preparation of GQD-doped GaN

The doping with GQDs was performed by a drop-cast method in the solvent of methanol. A 5 μL of drops of the as-prepared GQDs was dispensed onto the GaN surface by a pipette. The concentration of the GQDs per drop was 0.3 mg/ml. For higher concentrations, successive doping was carried out by repeating the same drop-cast steps and keeping the same amount of the GQD drop.

### Material characterization

The structural and chemical properties of the synthesized GQDs were analyzed using a transmission electron microscopic (TEM), X-ray photoelectron spectroscopy (XPS), and a micro Raman spectroscopy. The PL and photoconduction measurements were performed after the sample dried on a heater at 50 °C for 5 min. The excitation source in PL was used by a solid-state pulsed laser with the wavelength of 260 nm. The average power of the laser was kept as low as 0.5 mW to prevent from heating. The collected luminescence was projected into a 0.75 m spectrometer and detected using a high-speed photomultiplier tube (PMT). Time-resolved PL were carried out by the technique of time-correlated single-photon counting (TCSPC). The instrument response of the TCSPC system is about 200 ps. The I–V characteristics and photoconduction measurements of GaN epilayers were measured with a source meter (Keithley-2400) under a laser with the wavelength of 260 nm. The work function measurements of GQDs and GaN epilayers were carried out by the Kelvin probe measurement.

## Additional Information

**How to cite this article**: Lin, T. N. *et al.* Photo-induced Doping in GaN Epilayers with Graphene Quantum Dots. *Sci. Rep.* **6**, 23260; doi: 10.1038/srep23260 (2016).

## Supplementary Material

Supplementary Information

## Figures and Tables

**Figure 1:**
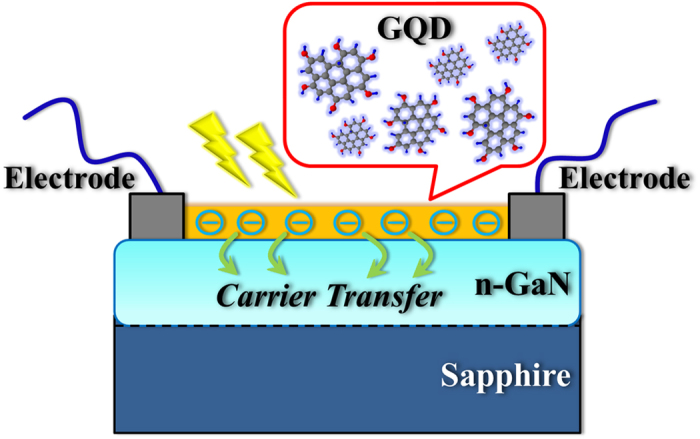
Schematic representation of the photoinduced doping system. The arrows represent the carrier transfer from graphene quantum dots (GQDs) to the GaN epilayer.

**Table 1:** The dispersion components β, the decay rates *k*, the PL decay times of the GQD-doped GaN versus the GQD concentration.

Concentration (mg/ml)	*β*	*k*(ns^−1^)	<*τ*> (ns)
0	0.57	5.55	0.29
0.3	0.55	3.85	0.44
0.6	0.52	2.56	0.73
0.9	0.51	2.04	0.98
1.2	0.50	2.27	0.88

**Table 2:** The dispersion components β, the decay rates *k*, the PL decay times of the GQDs after doping versus the GQD concentration.

Concentration (mg/ml)	*β*	*k*(ns^−1^)	<*τ*> (ns)
0	0.57	1.33	1.21
0.3	0.56	2.08	0.80
0.6	0.55	2.94	0.58
0.9	0.55	3.57	0.48
1.2	0.54	3.33	0.53

**Table 3:** The rise time constant *τ*_*r*_ and decay time constant *τ*_*d*_ used in the exponential fits.

	*τ*_*r*1_ (s)	*τ*_*r*2_ (s)	*τ*_*d*1_ (s)	*τ*_*d*2_ (s)
GaN	0.05	2.98	0.15	2.87
GQD/GaN	0.05	0.25	0.07	1.41
