# Supplementary material for: Photo-induced Doping in GaN Epilayers with Graphene Quantum Dots
Source: Sci Rep. 2016 Mar 18;6:23260. doi: 10.1038/srep23260 (PMC4796870; doi:10.1038/srep23260)
Supplement: Supplementary Information [file srep23260-s1.pdf]

Photoinduced Doping in GaN Epilayers with Graphene Quantum Dots

T. N. Lin[1], M. R. Inciong[1], S. R. M. S. Santiago[1], T. W. Yeh[1], W. Y. Yang[1], C. T. Yuan[1]*, J. L. Shen[1]*, H. C. Kuo[2] & C. H. Chiu[3]

[1]Department of Physics and Center for Nanotechnology, Chung Yuan Christian University, Chung-Li, Taiwan

[2]Department of Photonic and Institute of Electro-Optical Engineering, National Chiao Tung University, Hsin-Chu, Taiwan

[3] Department of Electronic Engineering, Chung Yuan Christian University, Chung-Li, Taiwan

*Corresponding author: ctyuan@cycu.edu.tw (Chi-Tsu Yuan), jlshen@cycu.edu.tw (Ji-Lin Shen)

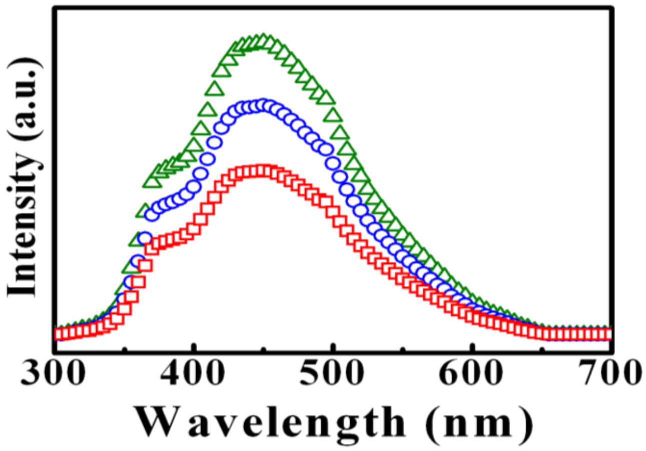

Figure S1. The photoluminescence spectra of graphene quantum dots (GQDs) with different GQD concentrations: 0.3 (open squares), 1.5 (open circles), and 2.4 mg/ml (open triangles).

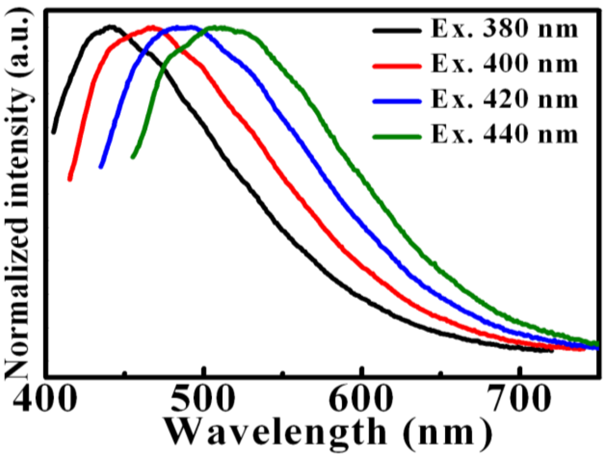

Figure S2. Photoluminescence spectra of GQDs with varying excitation wavelengths.

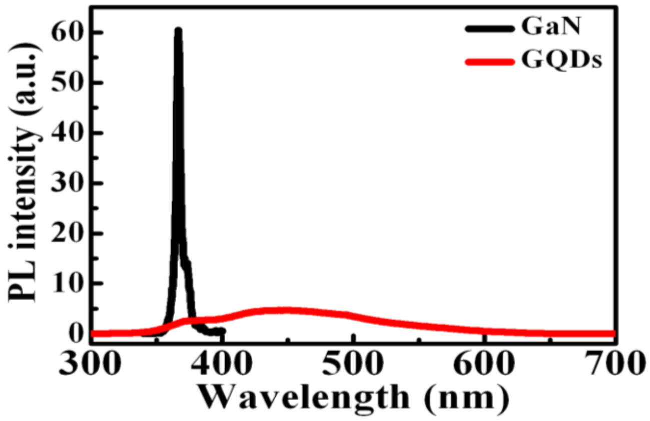

Figure S3. Photoluminescence spectra of GaN and GQDs when excited at a wavelength of 260 nm.

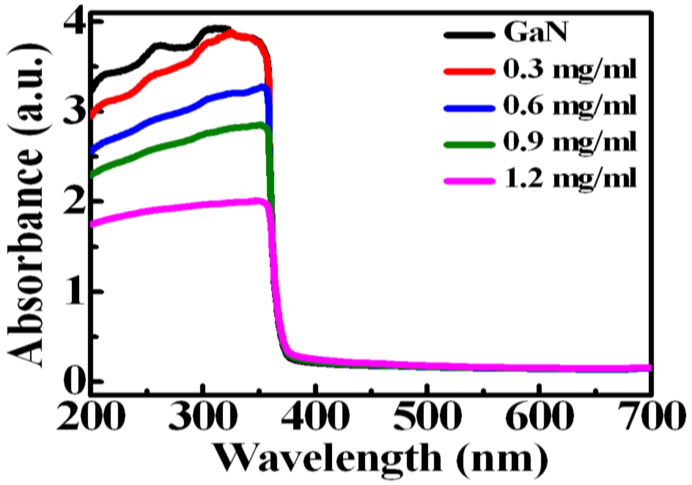

Figure S4. Absorption spectra of GaN with different GQD concentrations.
